# Supplementary material for: Enhancing surveillance of sexually transmitted infections in England with gender identity and behavioural data: The GUMCAD STI Surveillance System
Source: PLoS One. 2026 Jan 23;21(1):e0341128. doi: 10.1371/journal.pone.0341128 (PMC12829792; doi:10.1371/journal.pone.0341128)
Supplement: S1 Appendix — (DOCX) [file pone.0341128.s001.docx]

**Supporting Information (S1) – Appendix**

Appendix Table 1a – Variables included in the piloted GUMCAD Sexually Transmitted Infection Surveillance System, England (first pilot – 2013/14)

| **Sexual behaviour** | | |
| --- | --- | --- |
| *Heterosexual partnerships* | *Men who have sex with men* | *Women who have sex with men* |
| Number of heterosexual sex partners in the last 3 months? | Number of MSM sex partners in the last 3 months? | Number of WSW sex partners in the last 3 months? |
| ^†^How many of these were new sex partners (i.e. you have not had sex with them before)? | ^†^How many of these were new sex partners (i.e. you have not had sex with them before)? | ^†^How many of these were new sex partners (i.e. you have not had sex with them before)? |
| ^†^How many of these new sex partners were encounters abroad (i.e. you did not have sex in the UK)? | ^†^How many of these new sex partners were encounters abroad (i.e. you did not have sex in the UK)? | ^†^How many of these new sex partners were encounters abroad (i.e. you did not have sex in the UK)? |
| ^†^Were any of these encounters abroad with someone born in the: (please check all that apply) – UK, Western Europe, Eastern Europe, Sub-Saharan Africa, Caribbean, Other/Unknown Region | ^†^Were any of these encounters abroad with someone born in the: (please check all that apply) – UK, Western Europe, Eastern Europe, Sub-Saharan Africa, Caribbean, Other/Unknown Region | ^†^Were any of these encounters abroad with someone born in the: (please check all that apply) – UK, Western Europe, Eastern Europe, Sub-Saharan Africa, Caribbean, Other/Unknown Region |
|  | ^†^Of all the sex partners (in the last 3 months) how many did you have (receptive or insertive) anal sex with? |  |
|  | ^†^How many anal sex partners did you have unprotected (receptive or insertive) anal sex with? |  |
|  | ^†^How many unprotected anal sex partners were of a discordant or unknown HIV status? |  |
|  | ^†^How many unprotected anal sex partners did you have receptive anal sex with? |  |
|  | ^†^How many of these partners (you had unprotected receptive anal sex with) were living with HIV? |  |
|  | ^†^How many of these partners (you had unprotected receptive anal sex with) were HIV negative? |  |
| **Sexual behaviour** | | |
| Did you/your partner use a condom the last time you had penetrative (vaginal or anal) sex? | | |
| ^†^Were you under the influence of alcohol (before or during sex) with any partner in the last 3 months? | | |
| ^‡^Have you used recreational drugs in the last 6 months? | | |
| Were you under the influence of recreational drugs (before or during sex) with any partner in the last 3 months? | | |
| ^‡^Which drug did you take? (please check all that apply; responses : yes (injected/yes (not injected)/no) – Amphetamine/Speed, Cannabis, Cocaine, Crack, Crystal Meth/Methamphetamine, Ecstasy (E)/MDMA, GHB/GBL, Heroin, Solvents/Glue, Ketamine, Mephedrone (M-Cat), Methadone, Benzodiazepines (non-prescribed), Poppers, Other | | |
| **Previous STIs** | | |
| Have you ever attended another genitourinary medicine service – No, In the last year, In the last 1-5 years, Over 5 years ago | | |
| ^†^Are you living with HIV? | | |
| ^†^Are you living with Hepatitis C? | | |
| New registrants only: Have you been diagnosed with an STI in the past year? If so (check all that apply) – chlamydia, gonorrhoea, herpes (genital), LGV, syphilis, warts (genital), other  When did you last have an HIV test? – In the last year, In the last 1-5 years, Over 5 years ago | | |
| **Partner Notification** | | |
| Date of initial PN discussion | | |
| How many partners were reported during the relevant 'look-back interval'* for the STI(s) diagnosed? | | |
| How many of these partners were contactable? | | |
| How many of these partners were reported by the index patient as having attended a sexual health service (level 1, 2 or 3) within 4 weeks of the initial PN discussion? | | |
| How many of these partners were verified*** by a HCW as attending a sexual health service (level 1, 2 or 3) within 4 weeks of the initial PN discussion? | | |

^†^ - removed from the specification for the second pilot

^‡^ - amended in the specification for the second pilot

* Look-back interval (based on STI in keeping with guidance from the British Association for Sexual Health and HIV

** A partner is defined as contactable if the index patient is able and willing to either inform the partner of the risk of infection themself, or willing to pass on contact details to the healthcare work for provider referral.

*** 'Verified' means confirming contact attendance by checking records in your own service, or by contacting other services where contacts may have attended

Table 1b – Variables included in the piloted GUMCAD Sexually Transmitted Infection Surveillance System, England (second pilot – 2015/16)

| **Sexual behaviour** | | |
| --- | --- | --- |
| *Heterosexual partnerships* | *Men who have sex with men* | *Women who have sex with men* |
| Number of heterosexual sex partners in the last 3 months? | Number of MSM sex partners in the last 3 months? | Number of WSW sex partners in the last 3 months? |
| How many of these were new sex partners (i.e. you haven't had sex with them before)? | How many sex partners did you have in the last 3 months? | How many of these were new sex partners (i.e. you haven't had sex with them before)? |
|  | Have you had anal (receptive or insertive) sex with a known partner living with HIV in the last 3 months? |  |
|  | Have you had any condomless anal intercourse in the last 3 months? |  |
|  | Have you had any receptive condomless anal intercourse in the last 3 months? |  |
| **Sexual behaviour** | | |
| Was alcohol use assessed? If so, was alcohol use assessed as problematic? | | |
| Have you used recreational drugs in the last 3 months? | | |
| Which drug did you take? (please check box where applicable) – Amphetamine/Speed, Benzodiazepines (non-prescribed), Cannabis, Cocaine, Crack, Crystal Meth/Methamphetamine, Ecstasy (E)/MDMA, GHB/GBL, Heroin, Ketamine, Novel psychoactive substances, Mephedrone (M-Cat), Methadone, Poppers, Solvents/Glue, Other | | |
| Did you inject any recreational drug in the last 3 months? If yes: Did you share equipment with anyone when injecting drugs? | | |
| Were you under the influence of recreational drugs (before or during sex) the last time you had sexual intercourse? | | |
| ^†^**Previous STIs** | | |
| ^†^Have you ever attended another sexual health service? | | |
| ^†^New registrants only: Have you been diagnosed with an STI in the past year? If so – chlamydia, gonorrhoea, herpes (genital), LGV, non-specific genital infection, syphilis, warts (genital), other  ^†^When did you last have an HIV test? – In the last year, In the last 1-5 years, Over 5 years ago | | |
| **Partner Notification** | | |
| Date of initial PN discussion | | |
| How many partners were reported during the relevant 'look-back interval'* for the STI(s) diagnosed? | | |
| How many of these partners were contactable? | | |
| How many of these partners were reported by the index patient as having attended a sexual health service (level 1, 2 or 3) within 4 weeks of the initial PN discussion? | | |
| How many of these partners were verified*** by a HCW as attending a sexual health service (level 1, 2 or 3) within 4 weeks of the initial PN discussion? | | |
| ^†^How many of these partners were encounters abroad (i.e. you did not have sex in the UK)? | | |
| ^†^For people diagnosed with HIV or gonorrhoea: Were any of these encounters abroad with someone born in (select all that apply): Australasia, Caribbean, Eastern Europe, North America, South America, Southeast Asia, Sub-Saharan Africa, UK, Western Europe, Other | | |

^†^ - removed from the final GUMCAD specification

The current GUMCAD specification is published here: <https://www.gov.uk/government/publications/gumcad-clinical-guidelines>

* Look-back interval (based on STI in keeping with guidance from the British Association for Sexual Health and HIV

** A partner is defined as contactable if the index patient is able and willing to either inform the partner of the risk of infection themself, or willing to pass on contact details to the healthcare work for provider referral.

*** 'Verified' means confirming contact attendance by checking records in your own service, or by contacting other services where contacts may have attended

Appendix Table 2. Number of records submitted by sexual health services (SHS) that participated in the second pilot of the enhanced GUMCAD specification, England, 2015/16

| Clinic name | Number of records submitted |
| --- | --- |
| SHS 1 (East of England) | 963 |
| SHS 2 (South West) | 12,245 |
| SHS 3 (London) | 3,899 |
| SHS 4 (London) | 5,092 |
| SHS 5 (East of England) | 908 |
| Total | 23,107 |

Appendix Table 3. Feedback on the overall consistency, ease of completion, acceptability to patients, clarity of the technical guidance document and benefit of the proposed enhancement to GUMCAD, clinic staff from pilot sexual health services, 2016 (N=21)

| Variable | Agreed (%) | Not agreed (%) | Neutral (%) |
| --- | --- | --- | --- |
| Consistent with clinical practice | 43 | 19 | 38 |
| Ease of completion | 57 | 24 | 29 |
| Acceptable to patients | 71 | 10 | 19 |
| Clarity of the technical guidance document | 62 | 10 | 29 |
| Benefit of the proposed GUMCAD enhancement | 43 | 19 | 38 |

Source: Anonymous, self-administered online survey of clinicians who participated in the second pilot in 2015-16 (5 sexual health services participated in this pilot)

Appendix Table 4. Feedback on the ease of completion and usefulness of each of the four proposed behavioural domains of the enhanced GUMCAD specification, clinic staff from pilot sexual health services, 2016 (N=21)

|  | Response | Sexual behaviour (%) | Drug and alcohol use (%) | Previous STIs (%) | Partner notification (%) |
| --- | --- | --- | --- | --- | --- |
| Piloted variables were easy to complete |  |  |  |  |  |
|  | Yes | 81 | 48 | 48 | 10 |
|  | No | 10 | 33 | 33 | 0 |
|  | Not answered | 10 | 19 | 19 | 91 |
| Piloted variables were useful to collect |  |  |  |  |  |
|  | Yes | 81 | 57 | 62 | 10 |
|  | No | 5 | 19 | 14 | 0 |
|  | Not answered | 14 | 24 | 24 | 91 |

Source: Anonymous, self-administered online survey of clinicians who participated in the second pilot in 2015-16 (5 sexual health services participated in this pilot)

Appendix Figure 1. Survey instrument used to obtain feedback on the piloted enhanced GUMCAD specification after both the first and second pilots


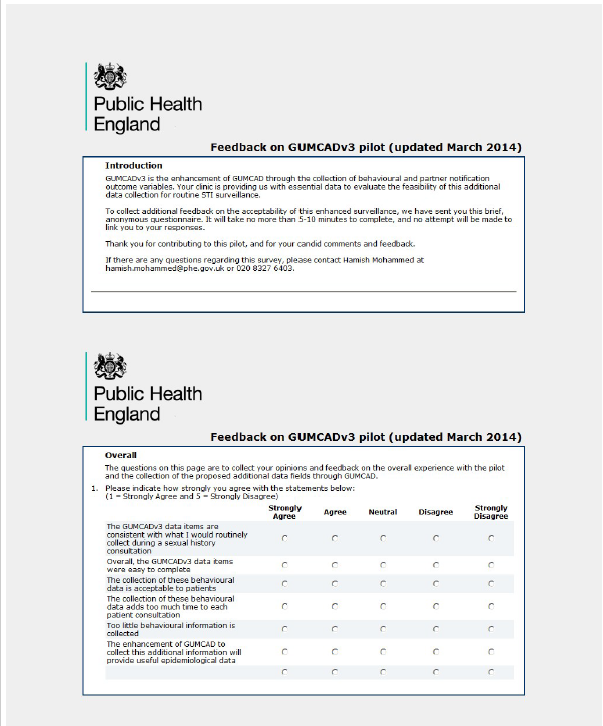


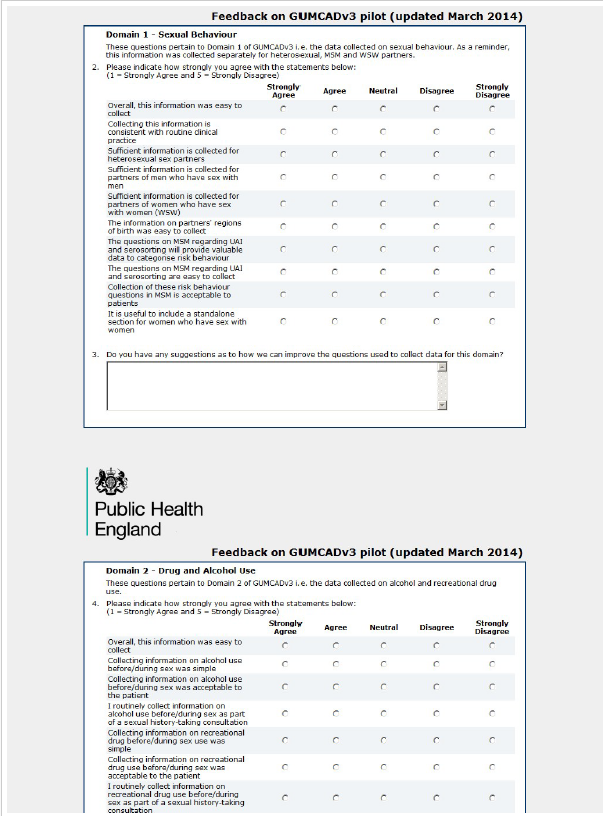

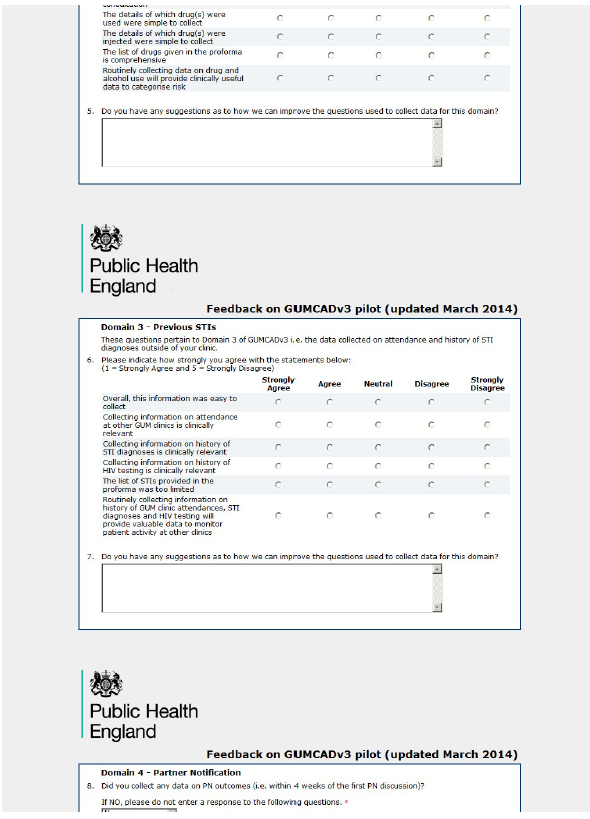


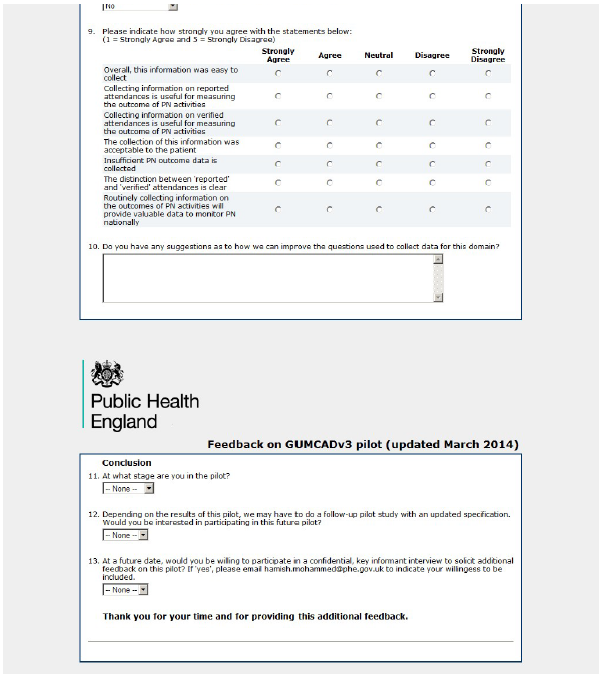


Notes: This survey was built in SelectSurvey. Public Health England is the predecessor to the UK Health Security Agency.

Appendix Figure 2. Proportion of patients attending 5 pilot sexual health services by gender and whether they had enhanced data submitted, pilot of enhanced GUMCAD specification, England, 2015/16

Key: GUMCADv3 – enhanced GUMCAD specification

The data collected in the pilot were based on a binary gender classification. The current version of GUMCAD collects data on gender identity (whether people are cisgender, transgender or gender-diverse).

Appendix Figure 3. Proportion of patients attending 5 pilot sexual health services by gender and age-group, and whether they had enhanced data submitted, pilot of enhanced GUMCAD specification, England, 2015/16

Key: Gv3 – enhanced GUMCAD specification

The data collected in the pilot were based on a binary gender classification. The current version of GUMCAD collects data on gender identity (whether people are cisgender, transgender or gender-diverse).

Appendix Figure 4. Proportion of patients attending 5 pilot sexual health services by gender and ethnicity, and whether they had enhanced data submitted, pilot of enhanced GUMCAD specification, England, 2015/16

Key: GUMCADv3 – enhanced GUMCAD specification

The data collected in the pilot were based on a binary gender classification. The current version of GUMCAD collects data on gender identity (whether people are cisgender, transgender or gender-diverse).

Appendix Document 1. Topic guide for key informant interviews of staff participating in the first pilot of the enhanced GUMCAD specification

*GUMCADv3 is the enhancement of GUMCAD through the collection of behavioural and partner notification outcome variables. Your clinic recently participated in a pilot to assess the feasibility of collecting these additional items, and I would like to get more of your thoughts on this, on how successful the pilot was, and how we could possibly improve it.*

*Request permission to audio-record*: **Y N**

Date (dd/mm/yy):___________

Clinic:_____________________

Position: ___________________

1. In your opinion, how did the pilot go at your clinic? Was there any resistance from staff?
   - Staff engagement
   - Impact on routine work
   - Patient engagement
   - Completion of proforma
2. Are the GUMCADv3 items usually collected during sexual history consultations? Were any questions awkward or difficult to ask?
3. Which questions/domains do you think worked well, and why?
4. Which questions/domains do you think did not work well, and why?
5. How did patients respond to being asked the questions:
   - On drug use
   - On risk behaviours (MSM online)
6. What do you think would be the utility of these data?
   - Feasibility of long-term implementation
   - Assessment of risk behaviours
