# Supplementary material for: Enhancing surveillance of sexually transmitted infections in England with gender identity and behavioural data: The GUMCAD STI Surveillance System
Source: PLoS One. 2026 Jan 23;21(1):e0341128. doi: 10.1371/journal.pone.0341128 (PMC12829792; doi:10.1371/journal.pone.0341128)
Supplement: Attachment — Submitted filename: Response to Reviewers.docx [file pone.0341128.s004.docx]

| **Point** | **Reviewer comment** | **Authors’ response** | **Action** | **Change in text (lines specified from continuous numbered lines in revised manuscript)** |
| --- | --- | --- | --- | --- |
|  | **Academic Editor** |  |  |  |
| 1 | Please ensure that your manuscript meets PLOS ONE's style requirements, including those for file naming. The PLOS ONE style templates can be found at | We have followed these style requirements. | All documents named in line with the style requirements | N/A |
| 2 | Thank you for stating the following financial disclosure: The first pilot was funded internally by Public Health England’s Innovation Fund. Public Health England is the predecessor of the UK Health Security (UKHSA). The first author and many coauthors are employees of UKHSA, the national health protection agency for England.  Please state what role the funders took in the study. If the funders had no role, please state: "The funders had no role in study design, data collection and analysis, decision to publish, or preparation of the manuscript." If this statement is not correct you must amend it as needed. Please include this amended Role of Funder statement in your cover letter; we will change the online submission form on your behalf. | We have amended the cover letter to state ‘PHE funded the pilots, and the salaries of all coauthors employed by PHE at that time’. Thank you for changing the online submission form on our behalf. | The cover letter has been amended to include the aforementioned statement. | N/A |
| 3 | Thank you for stating the following in the Competing Interests/Financial Disclosure section: AN was a member of the Steering Group for the pilots of the enhanced GUMCAD specification while he was an employee of Public Health England (PHE; the predecessor to the UK Health Security Agency [UKHSA]); he is now an employee of EpiConcept, a privately funded company. JW conducted analyses of the data collected from the pilots (the results of which are presented in the Appendix) while he was an employee of PHE; he is now an employee of Parexel International Limited, a privately funded clinical research organisation. None of the other coauthors reported any conflicts of interest.  We note that one or more of the authors are employed by a commercial company: EpiConcept and Parexel International Limited  a. Please provide an amended Funding Statement declaring this commercial affiliation, as well as a statement regarding the Role of Funders in your study. If the funding organization did not play a role in the study design, data collection and analysis, decision to publish, or preparation of the manuscript and only provided financial support in the form of authors' salaries and/or research materials, please review your statements relating to the author contributions, and ensure you have specifically and accurately indicated the role(s) that these authors had in your study. You can update author roles in the Author Contributions section of the online submission form.  Please also include the following statement within your amended Funding Statement. “The funder provided support in the form of salaries for authors [insert relevant initials], but did not have any additional role in the study design, data collection and analysis, decision to publish, or preparation of the manuscript. The specific roles of these authors are articulated in the ‘author contributions’ section.” If your commercial affiliation did play a role in your study, please state and explain this role within your updated Funding Statement.  b. Please also provide an updated Competing Interests Statement declaring this commercial affiliation along with any other relevant declarations relating to employment, consultancy, patents, products in development, or marketed products, etc.  Within your Competing Interests Statement, please confirm that this commercial affiliation does not alter your adherence to all PLOS ONE policies on sharing data and materials by including the following statement: "This does not alter our adherence to PLOS ONE policies on sharing data and materials.” (as detailed online in our guide for authors <http://journals.plos.org/plosone/s/competing-interests>) . If this adherence statement is not accurate and there are restrictions on sharing of data and/or materials, please state these. Please note that we cannot proceed with consideration of your article until this information has been declared.  Please include both an updated Funding Statement and Competing Interests Statement in your cover letter. We will change the online submission form on your behalf. | We have made the requested changes to the cover letter. Thank you for changing the online submission form on our behalf. | The following statements have been added to the cover letter:  EpiConcept and Parexel International provided support in the form of salaries for authors AN and JW respectively at the time of submission, but did not have any additional role in the study design, data collection and analysis, decision to publish, or preparation of the manuscript. Both AN and JW were employed by Public Health England (PHE) at the time of the pilot.  The specific roles of these authors are articulated in the ‘author contributions’ section.”  This does not alter our adherence to PLOS ONE policies on sharing data and materials. | N/A |
| 4 | We noted in your submission details that a portion of your manuscript may have been presented or published elsewhere. Please clarify whether this conference proceeding was peer-reviewed and formally published. If this work was previously peer-reviewed and published, in the cover letter please provide the reason that this work does not constitute dual publication and should be included in the current manuscript. | Some of the data (figures 1 and 2) were published with England’s annual sexually transmitted infection statistics. These results have not been peer-reviewed, nor have they been published elsewhere in conference proceedings or another journal. As a result, we believe this does not constitute dual publication – but please do advise of us if further information is required.  In our original version of the article, we cited the published data in the Results section (line2 258-259). | We have amended the cover letter as requested. | N/A |
| 5 | We note that you have included the phrase “data not shown” in your manuscript. Unfortunately, this does not meet our data sharing requirements. PLOS does not permit references to inaccessible data. We require that authors provide all relevant data within the paper, Supporting Information files, or in an acceptable, public repository. Please add a citation to support this phrase or upload the data that corresponds with these findings to a stable repository (such as Figshare or Dryad) and provide and URLs, DOIs, or accession numbers that may be used to access these data. Or, if the data are not a core part of the research being presented in your study, we ask that you remove the phrase that refers to these data. | We note this requirement and have deleted ‘data not shown’. | Mention of ‘data not shown’ has been deleted from lines 218 and 240. | Lines 218 and 240 |
| 6 | Please remove all personal information, ensure that the data shared are in accordance with participant consent, and re-upload a fully anonymized data set. | We have not uploaded a dataset given the advice from our Office for Data Acquisition and Release.  The data that support the findings of this study have been assessed by the UK Health Security Agency’s Office for Data Acquisition and Release as having sensitive personal information and are therefore not publicly available to protect participant privacy. However, some aggregate data may be available upon reasonable request from the UKHSA. Requests can be directed to [DataAccess@ukhsa.gov.uk](mailto:DataAccess@ukhsa.gov.uk) (UKHSA REGG Ref 524). | None | N/A |
| 7 | If the reviewer comments include a recommendation to cite specific previously published works, please review and evaluate these publications to determine whether they are relevant and should be cited. There is no requirement to cite these works unless the editor has indicated otherwise. | We note this recommendation. | None | N/A |
| Reviewer #1 | | | | |
| 1 | OVERALL In the results you highlight: "[In 2023,] transgender women, transgender men, and gender diverse (identifying as non-binary or in any other way) people each made up less than 1% of people accessing SHS in England". Given the comprehensiveness and reach of the GUMCAD tool, and considering the scarcity of representative estimates for trans and gender-diverse populations, this paper — and the journal's readership — would benefit from more prominent presentation of the following prevalence figures for SHS attendees: transgender women, 0.4%; transgender men, 0.5%; and gender-diverse individuals, 0.5%. | We agree and have made this edit. | The text has been amended ‘That year, transgender women (0.4%), transgender men (0.4%) and gender-diverse individuals (0.5%; people identifying as non-binary or in any other way)’ | Lines 268-271 |
| 2 | ABSTRACT a. Unless incidence estimates have been published elsewhere, and given the absence of clear evidence for increases in HSV or HPV, I suggest rephrasing to: “...an increasing trend in diagnosed bacterial sexually transmitted infections.”  b.Even within the abstract, it would be more accessible to spell out GUMCAD in full upon first mention. | a. We agree and have made this edit for clarity. b. We have defined the GUMCAD acronym. | a. The word ‘bacterial’ has been added. b. The GUMCAD acronym has been defined | a. Line 29 b. Line 31 |
| 3 | INTRODUCTION a. The opening paragraph comes across as somewhat dramatic. A large proportion of diagnosed bacterial STIs affect the rectum or oropharynx and often resolve spontaneously without leading to the sequelae described. Consider toning this down slightly.  b. P3L63: The second part of this sentence — suggesting a greater proportional rise among heterosexuals than MSM — is potentially misleading. For example, a rise from 1 to 2 cases among heterosexuals (100% increase) is proportionally greater than a rise from 5,000 to 6,000 among homosexual men (20%). Unless underpinned with absolute numbers, I suggest deleting the second half of the sentence.  c. P4L83: Reference 10 (Stutterheim et al.) reports on HIV prevalence, not "poorer sexual health." Among trans masculine individuals, the increased HIV risk appears primarily linked to behaviour — particularly sex with other men — rather than gender identity per se. I recommend rephrasing: “Given evidence for higher HIV prevalence among transgender men and particularly transgender women, when compared to the general population (10),...” | a. We note the reviewer’s concern but would still like to emphasise the harms and sequelae associated with STIs. b. We agree and have edited the text. c. We agree and have made this edit, and also added another related to STIs other than HIV. | a. None taken b. The text now reads ‘there has been a rapid resurgence in gonorrhoea [6] particularly among heterosexuals’ c. Text updated to ‘Additionally, given evidence of higher HIV prevalence among transgender men and particularly transgender women and high prevalence of other STIs in transgender people (compared to cisgender people) [13,14]’ | a. N/A b. Line 78 c. Lines 97-100 |
| 4 | METHODS a. P4L90: The sentence is quite long. Consider placing the legal clause (“under Regulation 4 of The Health Service – Control of Patient Information”) in parentheses or splitting it into a new sentence for readability.  b. P4L93: The term “Caldicott Advisory Panel” may be unfamiliar to international readers. I suggest adding a brief explanation such as: “...the UK's Caldicott Advisory Panel (named after Dame Fiona Caldicott).”  c. P5L106: The term “lower super output area” misses a word and should be capitalised and spelled out as: “Lower Layer Super Output Area (LSOA)”, which is likely more recognisable internationally.  d. P7L167: It would be more accurate to refer to “NHS England’s Data Coordination Board (DCB)” or, if discussing 2023 specifically, “formerly NHS Digital’s DCB”, as NHS Digital was dissolved and merged into NHS England in 2023. Consider clarifying this transition. Also, “NHS England Digital” may not be meaningful to non-UK audiences — best to avoid or explain. | a. We note this but have opted to retain the text to use the usual convention referring to this legislation and how we refer to analyses of surveillance datasets in the scientific literature. b. We agree this is an esoteric reference so have given additional context. c. We agree and have made this edit. d. We have amended the text for clarity. | a. No change made. b. The text has been amended as follows: ‘As approved by UKHSA’s Caldicott Advisory Panel, a group of information governance experts whose remit includes assessing whether data collections constitute public health surveillance activities,’ c. The text has been amended: ‘and lower-layer super output area of residence’ d. The text has been amended ‘The steering group endorsed the final version of the enhanced GUMCAD specification, after which UKHSA sought approval for this specification from the national Data Coordination Board (DCB)’ | a. N/A b. Lines 117-119 c. Line 133 d. Line 194 |
| 5 | RESULTS a. P8L203 and P10L260: Replace hyphens with en dashes (–) where ranges are indicated (e.g. “57–71%”).  b. P10L238: Add “in” before “96%”: “…gender identity was reported in 96% of records...”    c. P10L259f: The current wording is ambiguous. It could be read as implying that people with only one recent sexual partner had more STIs than those with multiple partners. If I understand correctly, partner number data were available for only 28% of cases in 2023. Suggested rephrasing: “In absolute numbers, heterosexual or bisexual women accounted for far more screens and attendances than heterosexual men. Where data on recent partner numbers were available, these women and men typically reported only one sexual partner in the past three months. In contrast, among GBMSM — who more frequently reported multiple partners — most screens and attendances involved men with 2–4 or 5+ recent partners.”  d. P11L271 and Fig. 1A: The current labelling mixes sexual identity and behaviour. Labelling only MSM by sexual identity while other groups remain behaviourally defined is inconsistent and could be read as stigmatising. A more consistent terminology might be: -HMSW (heterosexual and other men who have sex exclusively with women) -HBWSM (heterosexual, bisexual, and other women who have sex with men) -GBMSM (gay, bisexual, and other men who have sex with men) -LWSW (lesbian and other women who have sex exclusively with women)  In any case, where “MSW” is defined, clarify that it refers to “men who have sex exclusively with women.”  Since you mention that “women” in the figure include trans women, please also note the implications: for example, trans women with a penis having sex with each other are categorised as WSW. While this classification may be politically correct, it has implications for epidemiological reporting, particularly if the proportion of trans women increases in the future.  e. P11L279: Replace the hyphen with an em dash (—). | a. Having checked the journal’s submission guidelines, we have not made this change to avoid introducing any conflicts at the copyediting stage (should the article be accepted). b. We agree that this should be clarified. c. We agree and have made this edit. d. We agree with clarifying that ‘MSW’ refers to ‘men who have sex with women exclusively’. We understand the reviewer’s point but have retained the terminology on the original draft to facilitate comparisons with our grey and scientific literature publications, and to minimise the risk of confusion. We have used clinical and community stakeholder feedback to inform these terms, but agree with clarifying that ‘MSW’ refers to ‘men who have sex with women exclusively’.   We have also mentioned the point regarding transgender women as a limitation.   e. Having checked the journal’s submission guidelines, we have not made this change to avoid introducing any conflicts at the copyediting stage (should the article be accepted). | a. None b. The text has been amended: ‘gender identity was reported for 96% of records’ c. The text has been amended: ‘In absolute numbers, heterosexual or bisexual women accounted for far more screens and attendances than heterosexual men. Where data on recent partner numbers were available, these women and men typically reported only one sexual partner in the past three months. In contrast, among GBMSM — who more frequently reported multiple partners — most screens and attendances involved men with 2–4 or 5+ recent partners d. The text has been amended: ‘MSW – men who have sex with women (exclusively);’   We have added the following text ‘We acknowledge the limitation of aggregating cisgender and transgender women in some presentations of GUMAD data as the route of STI transmission cannot be inferred if transgender women with penis are classified as WSW’   e. None | a. N/A b. Line 267 c. Lines 286-291 d. Line 309   Lines 389-392   e. N/A |
| 6 | DISCUSSION a. P12L310: Add “diagnosed” before “bacterial” to reflect the fact that most countries (unlike the UK) lack comprehensive population-level testing data.  b. P13L324: See previous point. GBMSM includes gay, bisexual, and other men who have sex with men, many of whom may not identify as gay or bisexual. You could rephrase as: “MSM were more likely to report multiple sexual partners than MSW, WSM, or WSW,” or: “Gay and bisexual men were more likely to report multiple sexual partners than heterosexual men and women or lesbian women.”  c. P13L328: Please add “from three anatomical sites” after “...recommended to test for STIs quarterly”, to clarify the testing intensity involved, which impacts diagnosis rates.  d. P14L357: It is reassuring to read that the primary aim of using doxyPEP as a public health intervention is targeted at syphilis. Thank you for this. | a. We agree and have made this more explicit. b. We agree and have made this clearer as suggested. c. We agree and have made this edit. d. We note the reviewer’s comment. | a. Text has been amended as follows: ‘England, in common with other high-income countries, has detected an increase in bacterial STI diagnoses since the end of lockdowns’ b. The text has been amended ‘GBMSM were more likely to report multiple partnerships than heterosexual men and women or lesbians’ c. The text has been amended: ‘GBMSM (who are recommended to test for STIs quarterly from three anatomical sites’ d. None taken. | a. Line353 b. Lines 366-367 c. Line 370 d. N/A |
| Reviewer #2 | | | | |
| 1 | This paper represents important work in enhancing the surveillance system. The aim of the paper is somewhat unclear, however. There is some description of the consultation process to inform the enhancement work, though the detail of this is difficult to follow at time (important detail is left in supporting information). The GUMCAD data presented is limited in terms of responding to the main issues raised in the introduction. Further, the large proportion of missing behavioural data (i.e., gaps in number of recent sex partners) does not seem to support the statements about more robust behavioural data in the discussion. | We thank the reviewer for their feedback and have made several edits throughout the paper in response to clarify the aim, and to better frame the benefits of the reporting of these new behavioural data.. | The aim of the paper has been clarified and updated to specifically mention ‘The aim is this article is to describe and evaluate the impact of the piloting and rollout of an enhanced specification of GUMCAD at SHS in England’; this is reflected in the Abstract and the end of the Introduction. The abstract has also been updated substantially in light of this feedback.  Further edits have been made through the paper – including the start of the Discussion, which acknowledges the poorer completion of recent sex partner data.  The original draft acknowledged the need to minimise item non-response, but it now elaborates on this and mentions that the gender identity data were better completed than the recent sex partner data.  Lastly, the conclusion also acknowledged the need for further development to improve this data collection. | Lins 31-57  Lines 98-99  Lines 309-313  Lines 373-375  Lines 388-390 |
